# A preliminary metagenomics study of bacteria present in the dirt of Swiftlet farmhouses based on nitrite levels in edible bird’s nest on Sumatera Island, Indonesia

**DOI:** 10.14202/vetworld.2022.1798-1803

**Published:** 2022-07-26

**Authors:** Platika Widiyani, Mirnawati B. Sudarwanto, Hadri Latif, Denny Widaya Lukman, Daniel Thong, Puji Rahayu

**Affiliations:** 1 Indonesia Agriculture Quarantine Agency, South Jakarta, Indonesia; 2 Department of Veterinary Public Health, Faculty of Veterinary Medicine, IPB University, Bogor, Indonesia; 3 The Nusantara Farmers Edible Bird’s Nest Association, Tangerang, Banten, Indonesia; 4 Quality Control Laboratory and Certification for Animal Products, Bogor, Indonesia

**Keywords:** analysis, edible bird’s nest, metagenomic, nitrite, Swiftlet

## Abstract

**Background and Aim:**

Since the past decade, metagenomics has been used to evaluate sequenced deoxyribonucleic acid of all microorganisms in several types of research. Nitrite contamination originates from the natural environment in Swiftlet farmhouses (SFHs) and can influence nitrite levels in edible bird’s nest (EBN). It is strongly speculated that the conversion process into nitrite is influenced by the bacteria present in SFHs. Nitrite can cause adverse effects on human health. The previous research has focused on the characteristics of bacteria that may influence the nitrite conversion process in SFHs. This study aimed to a metagenomics analysis of bacteria present in the dirt of SFHs and evaluated nitrite levels in EBN on Sumatera Island.

**Materials and Methods:**

In total, 18 SFHs on Sumatera Island were selected, and EBN and dirt samples were collected from each SFH, resulting in 18 EBN and 18 dirt SFH samples. Raw uncleaned white EBN and dirt from three areas of SFH were collected. The samples were analyzed for nitrite levels using a spectrophotometer, and the metagenomics sequencing of SFH dirt samples was performed using the MinIon nanopore method. The sequenced data were analyzed using the EPI2ME software.

**Results:**

Of the 18 raw uncleaned white EBN samples, 9 (50%) had <30 ppm nitrite levels. The top five bacterial genera in SFH dirt samples in Group A (nitrite levels >30 ppm) were *Aeromonas*, *Escherichia*, *Acinetobacter*, *Arcobacter*, and *Acetoanaerobium*. Those in Group B (nitrite levels <30 ppm) were *Aeromonas*, *Pseudomonas*, *Shewanella*, *Escherichia*, and *Acinetobacter*. There were 12 genera of nitrifying bacteria in Group A and 8 in Group B. The total cumulative read of nitrifying bacteria in Groups A and B were 87 and 38 reads, respectively.

**Conclusion:**

This is the first study to show that characteristic bacteria present in the dirt of SFHs might significantly influence the conversion from nitrogen to nitrite. Approximately 50% of raw uncleaned EBN samples had <30 ppm nitrite levels. *Aeromonas* was the most dominant bacterial genus found in Groups A and B. The variations in genus and cumulative reads nitrifying bacteria in group A were greater than those in Group B. This study provides information on the characteristics of bacteria that may influence the nitrite conversion process in SFHs. Metagenomics data were obtained from the reading using the software EPI2ME. Further research is needed on the bacterial target species that can convert nitrite in SFHs.

## Introduction

Edible bird’s nest (EBN) is a food of animal origin produced from a pair of saliva glands of Swiftlets of the genus *Aerodramus* [1]. At present, Indonesia is the largest exporter and producer of EBN in the world [2]. Moreover, Indonesia is facing the challenge of providing high-quality EBN with low nitrite levels. The maximum limit for nitrite levels in EBN is 30 ppm, particularly for export to China [3], whereas regarding Decree of the Head of Indonesia National Standardization Agency No. 433 of 2021 stipulates the maximum limit for nitrite levels in EBN as 80 ppm [4]. EBN is a natural product and contains nitrite [5]. However, nitrite contamination in EBN can occur when the nest is still in its habitat. The formation of nitrite in EBN occurs through a natural process that involves changing nitrogen levels in the Swiftlet farmhouse (SFH) environment. Ammonia in SFH is oxidized to become nitrite and from nitrite may turn into nitrate. The conversion of nitrogen into nitrite is influenced by the bacteria present in SFH [5]. It is speculated that some bacteria can accelerate the process of nitrite formation in the environment. Metagenomics has been used to evaluate the sequenced deoxyribonucleic acid (DNA) of all microorganisms in several types of research [6–10]. Metagenomics allows for unbiased detection of organisms within a sample [9].

However, there are yet no data on the characterization of microorganisms in SFHs in Indonesia. Therefore, to the best of our knowledge, this is the first study, to perform metagenomics analysis to characterize and detect bacteria in the dirt of SFHs that are involved in nitrite formation. The study aimed to conduct a metagenomics analysis of bacteria found in the dirt of SFH based on nitrite levels in EBN on Sumatera Island, Indonesia.

## Materials and Methods

### Ethical approval

This study did not involve live swiftlets, so it did not require ethical approval.

### Study period and location

The study was conducted from August 2020 to October 2021. The EBN nitrite test was conducted in the Center of Diagnostic Standard of Agricultural Quarantine, Jakarta, and sequencing was taken in Quality Control Laboratory and Certification for Animal Products, Bogor.

### Sample collection

This study was conducted at 18 SFHs on Sumatera Island. Raw uncleaned white EBN, that is, white nest Swiftlet (*Aerodramus fuciphagus*) [11] and dirt of SFH were collected from each SFH. Three sampling areas in Sumatera Island, namely, A, B, and C, were selected. Area A has eight samples, Area B has six samples, and Area C has four samples. First, EBN samples were collected using a sterile spatula into food-grade plastic bags. Second, the dirt of SFH was collected aseptically using a sterile spatula into a plastic bag that had been disinfected. Third, the EBN samples were stored at 4°C for spectrophotometric analysis, and the dirt of SFH was stored in the laboratory at 0°C for metagenomics analysis. The criterion for collecting the EBN sample was raw uncleaned white EBN with a medium level of fur cleanliness. Approximately 1 or 2 g of raw uncleaned EBN per SFH was collected. The raw uncleaned EBN still contained hair and other impurities and required a cleaning process [12]. Dirt weighing approximately 50 g was collected from each SFH.

### Nitrite analysis

Nitrite analysis was conducted using a spectrophotometer as described previously [13] with several modifications. The analysis was performed using the following reagents: standard nitrite (Merck, Germany), sulfanilamide (Merck), and N-(1-naphthyl) ethylenediamine dihydrochloride (NED) solutions (Merck). The standard solution of nitrite (Merck) was diluted and mixed with 0.6 mL of saturated NaCl (Merck) and 9.4 mL of ion-free water (Millipore, Ireland). Next, 1 mL of sulfanilamide (Merck) was added and allowed to stand for 5 min, followed by the addition of 1 mL of NED (Merck). This solution was allowed to stand for 15 min, after which the absorbance was measured using a spectrophotometer (Thermo Fisher Scientific, USA). The EBN sample was homogenized for nitrite determination, and then 40 mL of ion-free water (Millipore) and 3 mL of saturated NaCl solution (Merck) were added. The mixed solution was heated in an ultrasonic digester (Thermo Fisher Scientific) at 40°C for 30 min and filtered using Whatman paper no. 41 (Whatman, UK). Next, 2.5 mL of sulfanilamide (Merck) was added and allowed to stand for 5 min. Subsequently, 2.5 mL of NED (Merck) was added, homogenized, and allowed to stand for 15 min. Then, the absorbance was measured using a spectrophotometer at a wavelength of 541 nm. Moreover, the concentration of nitrite levels is calculated according to the calculation formula:


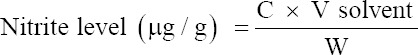


Where,

C = the amount of nitrite in the sample obtained from the calibrated curve (mg/L)

V = sample solvent volume (mL)

W = sample weight (g).

### Metagenomics analysis

Metagenomics analysis was performed using Oxford Nanopore Technologies (ONT) MinION (Oxford, UK). This analysis consisted of three stages, namely, SFH dirt sample preparation, sample extraction, and testing using MinION. The SFH dirt samples were prepared and added to 600 mL of nuclease-free water (Qiagen, Germany) and then filtered using a vacuum filter (Whatman) coated with a nitrocellulose filter (NF) membrane (Millipore). The NF membrane was extracted using a standard procedure of the Qiagen DNeasy PowerWater kit (Qiagen) and ONT SQK-RBK004 Rapid Barcoding Kit (Oxford). DNA quantification was done using a Nanodrop spectrophotometer to measure the concentration and purity of DNA. The DNA concentration obtained using the nanodrop spectrophotometer (Thermo Fisher Scientific) must match the predetermined value of 1.8–2.0 (A260/A280). DNA sequencing requires approximately ± 400 ng of DNA. Then, the DNA was processed using the ONT SQK-RBK004 Rapid Barcoding Kit (Oxford), wherein a 75-μL premix was obtained that was inserted into the flow cell on the ONT MinION by dropping slowly and each drop flowed into the port before adding the next drop. In the next stage, starts running the MinKNOW program on a computer or laptop that has been connected to the MinION ONT until sequence data obtain in the form as Fastq file. Data were obtained as Fastq file and then analyzed using Epi2ME (Oxford) to obtain bioinformatics data [6].

### Statistical analysis

The formation of nitrite was analyzed using Microsoft Excel (Microsoft Office, USA). The metagenomics data were analyzed using Epi2ME (Oxford) and represented as figures using Microsoft Excel.

## Results

The results of nitrite analysis showed that of the 18 raw uncleaned EBN samples, 9 (50%) had nitrite levels of <30 ppm. The average nitrite content in raw uncleaned EBN samples was 55.77 ppm, with the median value being 33.05 ppm (Table-1).

**Table 1:** Data on nitrite level in raw uncleaned white edible bird’s Nest from Sumatera Island.

Origin	number of Samples	Maximum Nitrite Level (ppm)	Minimum Nitrite Level (ppm)	Average of Nitrite Level In Each Area (ppm)	Total Average of Nitrite Level (ppm)	Median of Nitrite Level (ppm)
Area A	8	181.19	35.15	98,10	55.77	33.05
Area B	6	22.60	15.36	18.07		
Area C	4	36.71	19.46	27,92		

The nitrite levels in raw uncleaned EBN samples were used as a reference in the metagenomics analysis with a cutoff of 30 ppm, which resulted in the following two groups: Group A (nitrite levels >30 ppm) and Group B (nitrite levels <30 ppm). The cumulative reads analyzed sequentially in Groups A and B showed 524,719 and 672,000 bases, respectively. The average length of the sequences obtained in Groups A and B was 2955 bases and 1029 bases, respectively.

**Figure-1:**
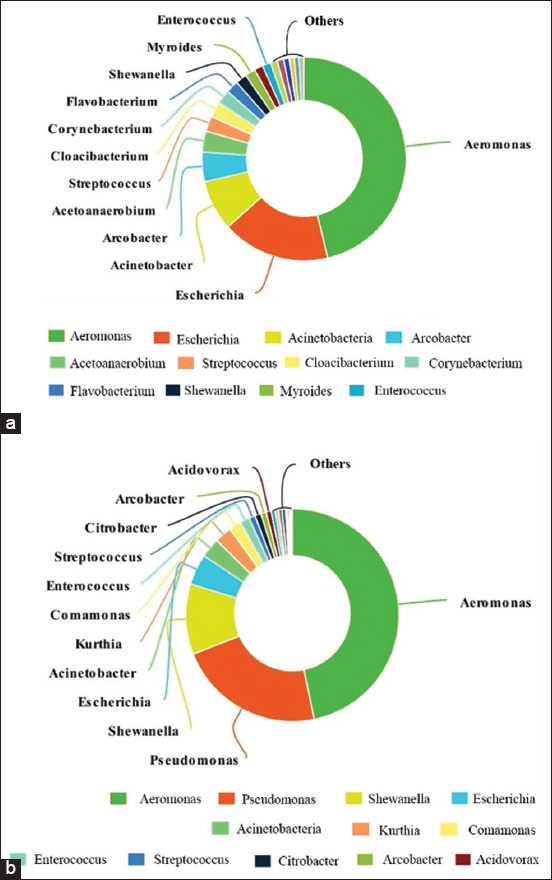
(a and b) The most dominant bacterial genus found in Groups.

This study identified a bacterial profile community based on the metagenomics analysis of SFH dirt samples. The five most abundant bacterial genera in Group A were *Aeromonas* (41.6%), *Escherichia* (15.4%), *Acinetobacter* (7.2%), *Arcobacter* (4.2%), and *Acetoanaerobium* (3%). In Group B, the most abundant bacterial genera were *Aeromonas* (45%), *Pseudomonas* (21.5%), *Shewanella* (10.3%), *Escherichia* (4.5%), and *Acinetobacter* (2.9%). *Aeromonas* was the most dominant bacterial genus found in both groups (Figure-1).

**Figure-2:**
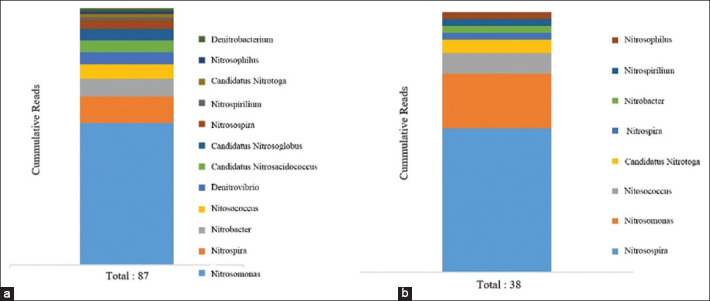
(a and b) The nitrifying bacteria in the dirt of Swiftlet farmhouses in Groups.

This study also identified nitrifying bacteria in the SFH dirt samples. The nitrifying bacteria found in Groups A and B showed differences in terms of the number of genera, that is, 12 genera were found in Groups A and 8 genera were found in Group B. The 12 genera of nitrifying bacteria found in Group A with cumulative reads sequentially were *Nitrosomona*s (48), *Nitrospir*a (9), *Nitrobacte*r (6), *Nitrosococcu*s (5), *Denitrovibri*o (4), *Candidatus Nitrosacidococcu*s (4), *Candidatus Nitrosoglobu*s (4), *Nitrosospir*a (3), *Nitrospirillu*m (1), *Candidatus Nitrotog*a (1), *Nitrosophilu*s (1), and *Denitrobacteriu*m (1) (Figure-2). The total cumulative reads of nitrifying bacteria were 87 reads in Group A. In Group B, the 8 genera of bacteria with cumulative reads sequentially were *Nitrosospira* (21), *Nitrosomonas* (8), *Nitrosococcus* (3), *Candidatus Nitrotoga* (2), *Nitrospira* (1), *Nitrobacter* (1), *Nitrospirillum* (1), and *Nitrosophilus* (1) (Figure-2). The total cumulative reads of nitrifying bacteria in Group B were 38 reads.

## Discussion

This study showed that the total average nitrite level in raw uncleaned white EBN samples was >30 ppm. There is yet no national or international regulation on the nitrite levels of raw uncleaned white EBN. The value is relatively high, but it could be because the samples were raw uncleaned EBN that were not washed or processed initially (6). Raw uncleaned EBN samples show wide variations in nitrite levels. The nitrite levels obtained in this study are different from those in cleaned EBN samples in the Hong Kong market, which showed nitrite level variations between 0 and 6430 ppm [5]. Different washing frequencies cause a reduction in nitrite levels. The nitrite level in EBN is influenced by the duration of exposure of EBN to water. The longer the EBN is exposed to water, the lower the nitrite level [5, 14].

High nitrite consumption causes digestive disorders, bloody diarrhea, chronic poisoning, and death. It can increase cancer risk factors due to carcinogenic N-nitrosamine compounds [15]. The nitrite content in food has been associated with methemoglobinemia in infants [16]. Nitrite levels in EBN are also influenced by the environmental conditions of SFH and EBN conditions, such as color, nest cleanliness, and nest age. Environmental factors can originate through the decay of organic material on the SFH floor [17]. The accumulation of high nitrite levels is influenced when Swiftlets build their nests [18]. Moreover, the formation of nitrite in EBN occurs through a natural process such as changes in nitrogen levels in the SFH environment [5].

Nevertheless, the limitation of the research related to the profile of all bacteria in SFH using MinION sequencing might be worthwhile as suggestions considering for future research shall be mentioned. First, bacterial profiles can be obtained using metagenomics analysis, in which the entire DNA of the microorganism community is analyzed through random sequencing and not just by sequencing specifically targeted genes [7]. The advantages of the latest generation of sequencing include fast reading times [8], providing unbiased detection of organisms in samples [9], displaying community diversity, and characterizing the composition of microorganisms [19]. The reading results produced by MinION are of high quality, and the resulting taxonomy is accurate at 99.5% [20]. Metagenomics analysis using MinIon utilizes EPI2ME to explore metagenomics data easily and provides more reliable information at family and genus levels [6]. These results are consistent with research on compost (feces and manure), wherein the number of nitrogen-fixing bacteria was increased significantly during the composting process in Uganda, and one of those bacteria was the genus *Aeromonas*. *Aeromonas* is the most dominant bacterial genus. It contributes a high total abundance percentage as assessed by PCR [21]. Manure comprises an ecosystem with the most diverse and interacting bacterial communities [22]. Reliable nanopore sequencing data can be used to classify communities of species and genera of bacteria and capture microbiota diversity in the sample [10].

Cleanliness of the SFH environment is a crucial factor to consider and strongly correlates with the amount of nitrite in EBN. It has been demonstrated that EBN can be contaminated with nitrite from the environment [14]. Nitrite is formed naturally by the oxidation of sodium nitrate (NaNO_3_) by nitrogen in the air. This nitrogen must be converted into ammonia and nitrite, and nitrite is converted into nitrate by nitrifying bacteria. The process of nitrification is divided into two stages. The first stage is the formation of nitrite (NO_2_), and the second stage is nitration, that is, the conversion from nitrite into nitrate form (NO_3_) [23, 24]. A study on Swiftlet feces and air conditions using 16S rRNA in an SFH of Sarawak, Malaysia, detected the gram-positive pathogenic bacteria *Bacillus*, *Lysinibacillus*, *Paenibacillus*, and *Sporosarcina* [25]. Analysis of the air samples of SFH in Malaysia using 16S rRNA sequencing revealed 27 species of airborne bacteria, with *Lysinibacillus* spp., being the most common. Air contains microorganisms such as bacteria, fungi, and viruses. Exposure to airborne bacteria can cause adverse human health effects [26].

The bacteria capable of converting ammonia into nitrite include the phylum Proteobacteria, specifically of the Beta class (e.g., *Nitrosomonas* and *Nitrosospira*) and Gamma class (*Nitrosococcus*) [19]. Nitrosomonadaceae are the dominant nitrite-forming bacteria in soil, namely, *Nitrosospira* (including *Nitrosovibrio* and *Nitrosolobus*) and *Nitrosomonas*. The most abundant nitrite-forming bacteria in soil metagenomics were *Nitrosospira* (50–80%), *Nitrosomonas* (13–41%), and gammaproteobacteria (<10%). These bacteria do not compete directly in the soil but occupy complementary niches [8]. *Nitrosomonas* are heterotrophic bacteria and produce the enzyme catalase, which plays a role in the process of ammonia oxidation to nitrite. *Nitrosomonas* and *Nitrosococcus* obtain energy by oxidizing ammonium carbonate. The oxidation process of ammonia to nitrite in the soil can also be mediated by *Nitrosospira* and *Nitrosomonas* bacteria or the crenarchaeum *Nitrososphaera*. In contrast, nitrite oxidation can be mediated by *Nitrobacter* and *Nitrospira* bacteria [23].

## Conclusion

The average nitrite level in raw uncleaned EBN samples collected from Sumatera was 55.77 ppm, with a median value of 33.05 ppm. Characteristic bacteria were detected in the dirt of SFH, which might affect the process of conversion from nitrogen into nitrite. *Aeromonas* was the most dominant bacterial genus found in the dirt samples of SFH in Group A (nitrite content >30 ppm) and Group B (nitrite content <30 ppm). The variations in genus and cumulative reads of nitrifying bacteria in Group A were higher than those in Group B. Metagenomics data were acquired based on the reading using EPI2ME. This study provides an overview of the types of bacteria found in SFHs, which could be useful for making environmental modifications to prevent bacterial growth in SFHs. However, further research is needed on the bacterial target species that can convert nitrite in SFHs.

## Authors’ Contributions

PW, MBS, HL, DWL, DT, and PR: Designed the study. PW: Collection of data. PW, MBS, HDL, DWL, and PR: Analyzed the data and drafted the manuscript. PW, MBS, HL, DWL, DT, and PR: Designed the study, analyzed the data, and drafted the manuscript. All authors have read and approved the final manuscript.
